# Fracture incidence in adults in relation to age and gender: A study of 27,169 fractures in the Swedish Fracture Register in a well-defined catchment area

**DOI:** 10.1371/journal.pone.0244291

**Published:** 2020-12-21

**Authors:** Camilla Bergh, David Wennergren, Michael Möller, Helena Brisby

**Affiliations:** 1 Institute of Clinical Sciences, Sahlgrenska Academy, Gothenburg University, Gothenburg, Sweden; 2 Department of Orthopaedics, Sahlgrenska University Hospital, Gothenburg, Sweden; Medical College of Wisconsin, UNITED STATES

## Abstract

Studies on fracture incidence have mostly been based on retrospectively registered data from local hospital databases. The Swedish Fracture Register (SFR) is a national quality register collecting data prospectively on fractures, at the time of care-seeking. In the present study the incidence of all different fractures, regardless of location, in adults’ ≥ 16 years treated at the only care provider for patients with fractures within a catchment area of approximately 550,000 inhabitants, during 2015‒2018 are described. Age, gender, and fracture location (according to AO/OTA classification) was used for the analyses and presentation of fracture incidences. During the 4-year study period, 23,917 individuals sustained 27,169 fractures. The mean age at fracture was 57.9 years (range 16‒105 years) and 64.5% of the fractures occurred in women. The five most common fractures accounted for more than 50% of all fractures: distal radius, proximal femur, ankle, proximal humerus, and metacarpal fractures. Seven fracture incidence distribution groups were created based on age- and gender-specific incidence curves, providing visual and easily accessible information on fracture distribution. This paper reports on incidence of all fracture locations based on prospectively collected data in a quality register. The knowledge on fracture incidence related to age and gender may be of importance for the planning of orthopaedic care, involving both in- and out-patients as well as allocating surgical resources. Further, this might be useful for organizing preventive measures, especially in countries with similar socioeconomic structure and fracture burden.

## Introduction

Fractures occur in individuals of all ages. However, the type and body location vary widely depending on different factors, mainly related to individual bone quality and the nature of the trauma. From a societal view it is of interest to know the incidence of different fractures in a certain population. Such knowledge can form a base for the organization of relevant healthcare and for undertaking preventive measures to mitigate the risk of fractures. This may involve general community organization, including the planning of road traffic and living conditions for the elderly, but even more specific preventive measures for certain risk groups [1–3]. Especially for the fractures occurring in the elderly population, which are often fragility fractures, increased preventive measures are of interest. The World Health Organization (WHO) has described fragility fractures as fractures that result from mechanical forces that would not normally lead to a fracture [4]. The incidence of fractures in many locations have been reported to increase [5–7], which mainly could be attributed to an increase in numbers of fragility fractures in a growing elderly population [8]. There are also reports of decreasing fracture incidences for certain fractures [9–11]. Many factors may contribute to changes in the incidence rates―such as comorbidities of diabetes, obesity, and others [11–13]; the use of certain medications; mental factor and social factors [14, 15].

The population above 50 years of age in Sweden is expected to increase by 18% between 2010 and 2025, and it has been estimated that the total number of fractures will increased by 26% during this period [16]. Until now, the calculation of fracture incidences has been based on local hospital databases [17] and to a lesser extent on national databases derived from hospital reports [5, 18]. For example, Driessen et al. reported a total fracture incidence of 1,910 per 10^5^ inhabitants per year based on the Danish National Hospital Discharge Register, and described the incidence according to age and gender [19]. To date, there has been no overview, to our knowledge, of fracture incidence based on national quality registers data; until now, the focus has been on one specific fracture location, by bone or segment, e.g. clavicle, tibia, or proximal humerus fractures [20–22]. Although not making use of a formalized national register, but instead basing their findings on the radiographic evaluation of almost 6,000 fractures seen at one hospital over one year, Court-Brown et al. (2006) reported on the epidemiology of adult fractures [23]. Prospectively collected data, as in a national quality register, can be expected to be more accurate―regarding both fracture location and type―than studies based on data from hospital databases.

In the Swedish Fracture Register (SFR), the data are collected prospectively for all in-patients and out-patients using a web-based platform. Initial registration is performed by the orthopedic surgeon/resident/intern that has the initial contact with the patient (usually at the Accident and Emergency Department). The patient-related data include type of trauma, location of fracture, and treatment―regardless of whether it is planned to be surgical or non-surgical.

Traditionally, low-energy fractures of the thoracolumbar vertebrae, pelvis, distal radius, proximal femur, and proximal humerus have been considered to be fragility fractures [24–27], but fractures in all segments of the tibia and in the distal femur may also be added to this group [21, 23]. In the study by Court-Brown et al., the fracture types were divided into eight different groups based on gender and age distribution. It was concluded that about one-third of fractures in men and two-thirds of fractures in women should be considered to be possible osteoporotic fractures [23].

The main aim of the present study was to determine fracture incidence in adults, prospectively collected in a quality register, with special focus on potential fragility fractures, and to produce incidence curves according to age and gender. Further, to divide fractures into groups, according to similarity in patterns of incidence regarding age and gender, to simplify the interpretation of the incidence figures.

## Material and methods

### Data collection in the Swedish Fracture Register

The SFR was started in 2011 with collection of data on fractures treated at Sahlgrenska University Hospital (SU) in Gothenburg, and the data collection procedure has been described in detail [28]. The SFR has expanded gradually and at the beginning of 2020 more than 95% of the orthopaedics departments in Sweden had been linked to the SFR [28]. Overall registrations, and selected data and results from the SFR are presented yearly in the annual report [29]. When the data from the SFR has been compared with the official national health database (the Patient Register at the Board of Health and Welfare), the completeness of registration of fractures has been between 70% and 95% for most participating departments [29].

Currently, about 80,000 fractures are registered annually in the SFR. All fractures, both surgically and non-surgically treated, are registered and classification is done using the Muller Arbeitsgemeinschaft für Osteosynthesefragen/ Orthopaedic Trauma Association (AO/OTA) classification system [30, 31]. Validation of the accuracy of the fracture classification in SFR has been done for fractures of humerus, tibia, ankle, femur and vertebral fractures [32–36]. Using Cohens kappa, values between 0.66–0.83 was achieved for AO/OTA fracture type and 0.56–0.67 for AO/OTA fracture group. According to Landis and Koch these values range from moderate to almost perfect agreement. The validation studies in SFR has shown that fractures classified mainly by junior doctors at the Accident & Emergency departments have the same level of accuracy as previous studies performed among senior experts only. Registration of trauma mechanism (low, high, unknown or undecidable) and whether the fracture is open or closed are performed at the primary patient registration. Classification of trauma mechanism was based on advice in the SFR user manual with reference to national trauma alert criteria, no systematic algorithm was used.

### The data set used in the present study

Data on all patients, 16 years of age and older, who were treated for a fracture at the trauma center at SU in Gothenburg between 1 January 2015 until 31 December 2018, were extracted from the SFR regardless of trauma mechanism. This is the only care provider within the catchment area treating fracture patients. All primary fractures but no re-fractures were included. This means that more than one fracture (different anatomical locations) could be registered for one patient at the same day or at different occasions during the four-year period included. For calculation of incidence and age-specific incidence, data for the population of Gothenburg and the surrounding municipalities (the catchment area of SU) were obtained from Statistics Sweden (data available online). The population of the catchment area of SU included 541,316 individuals in 2014 and increased to 564,335 individuals in 2018 (267,421‒280,829 men and 273,895‒283,506 women). The total number of person years were 2 210,619.

Inclusions were limited to adults, defined as individuals who were 16 years of age or older (the register does not have complete data for all fracture locations in individuals younger than 16 years of age). The fractures were divided into 27 anatomical regions according to the AO/OTA classification [30]. The fractures were grouped based on age and gender incidence curves, where attention was payed to their visual appearance and included factors such as peak incidence age, slope of the curves, and gender similarities/differences.

### Statistics

Incidence is reported numbered by 100.000 individuals. When calculating the incidence rate, the exposure time (person years) for each of the 4 years was approximated by the number of individuals in the population at a given date, and the total exposure time during the 4 years was calculated as the sum of the 4 years. For each age-group, gender and total, exact 95% confidence intervals for the incidence rate were calculated using the Poisson distribution. All statistical analyses for tables and figures were done using SAS (v9.4).

### Ethics

The present study was approved by the Central Ethical Review Board, Gothenburg (ID 792–17). All patients who are registered in the SFR receive information about their registration and are given the option of withdrawing. The data from SFR were fully anonymized before data analysis begun.

## Results

### Fracture incidence, overall, for different locations and in relation to gender

During the 4-year study period, 23,917 individuals sustained 27,169 fractures. The mean age at fracture was 57.9 years (range 16‒105 years) and 64.5% of the fractures occurred in women (Table 1).

**Table 1:** Descriptive statistics of gender and age for all sustained fractures.

Gender	Age * (years)					
	n	Mean	SD	Min	Median	Max
Male	10,132	48.7	22.1	16	46.0	102
Female	13,785	63.6	20.5	16	66.0	105
All	23,917	57.3	22.4	16	59.0	105

*Age is for one random injury in those with several injuries during the four years.

The overall incidence was 1,229 fractures per 100,000 individuals per year. This gives a person-yearly fracture incidence rate of 1.2%. The numbers and proportions of fractures, according to the bone affected, and for some according to a segment of the bone, are presented in Table 2. The most common fractures in the total population were located in the distal radius (n = 4,445, 16.4%), the proximal femur (n = 3,993, 14.7%), the ankle (n = 2,799, 10.3%), the proximal humerus (n = 2,237, 8.2%), and the metacarpal bones (n = 1,964, 7.2%). For all remaining fracture types, the proportions were less than 6%.

**Table 2:** Incidence of all fractures (2015–2018). Number of fractures sorted by location, proportion of fractures caused by high energy trauma, proportion of open fractures and incidence per 100,000 person-years (2015–2018) in total and by gender. Fractures arranged in order of decreasing incidence.

Fracture	n (%)	High-energy	Open fracture	Total:	Men:	Women:
		%	%	n/100,000	n/100,000	n/100,000
Distal Radius	4,445 (16.4)	5.3	1.6	201.1	108.2	292.4
Proximal femur	3,993 (14.7)	1.8	0.2	180.6	123.2	237.1
Ankle	2,799 (10.3)	4.5	1.6	126.6	100.3	152.5
Proximal humerus	2,237 (8.2)	4.1	0.4	101.2	59.2	142.5
Metacarpal	1,964 (7.2)	6.2	0.9	88.8	125.6	52.7
Finger phalanx	1,549 (5.7)	10.0	12.2	70.1	77.6	62.6
Proximal forearm	1,413 (5.2)	6.5	1.4	63.9	54.6	73.0
Metatarsal	1,339 (4.9)	7.0	0.7	60.6	50.0	71.0
Clavicle	991 (3.6)	27.0	0.2	44.8	61.9	28.1
Toe phalanx	804 (3.0)	5.6	6.4	36.4	32.6	40.1
Pelvis	716 (2.6)	15.2	0.5	32.4	16.4	48.1
Spine	670 (2.5)	22.2	0.0	30.3	32.4	28.3
Carpus	606 (2.2)	10.5	0.0	27.4	38.8	16.2
Proximal tibia	545 (2.0)	19.9	1.6	24.7	21.2	28.2
Humeral diaphysis	323 (1.2)	12.0	2.2	14.6	13.4	15.8
Patella	308 (1.1)	5.8	2.8	13.9	12.0	15.9
Scapula	304 (1.1)	27.9	0.7	13.8	17.4	10.1
Tibia diaphysis	291 (1.1)	25.5	16.3	13.2	14.9	11.5
Midfoot	283 (1.0)	34.9	1.5	12.8	13.8	11.8
Calcaneus	252 (0.9)	48.6	3.6	11.4	15.0	7.9
Distal humerus	249 (0.9)	12.5	8.5	11.3	7.8	14.7
Distal tibia	217 (0.8)	31.5	15.0	9.8	10.7	9.0
Femoral diaphysis	211 (0.8)	14.2	3.8	9.5	5.2	13.8
Distal femur	204 (0.8)	9.8	5.3	9.2	4.8	13.5
Forearm	196 (0.7)	27.4	14.4	8.9	9.9	7.9
Acetabulum	164 (0.6)	32.2	1.1	7.4	10.0	4.8
Talus	96 (0.4)	52.9	7.8	4.3	5.6	3.1
Total	27169 (100.0)	9.2	2.3	1229.0	1042.1	1412.8

n, number of fractures for each fracture type; %, percentage of all fractures.

For the total cohort 9.2% of the fractures were registered as had been caused by high-energy trauma, with a large variation between different fracture locations. The proportions of open fractures were 2.3% for all fractures (for details on the different fracture locations see Table 2).

Gender specific incidence for all fracture locations are given in Table 2. For both genders, distal radius and proximal femur fractures are among the three most common fractures.

### Detailed description of fracture incidence in relation to age and gender

**Fig 1:**
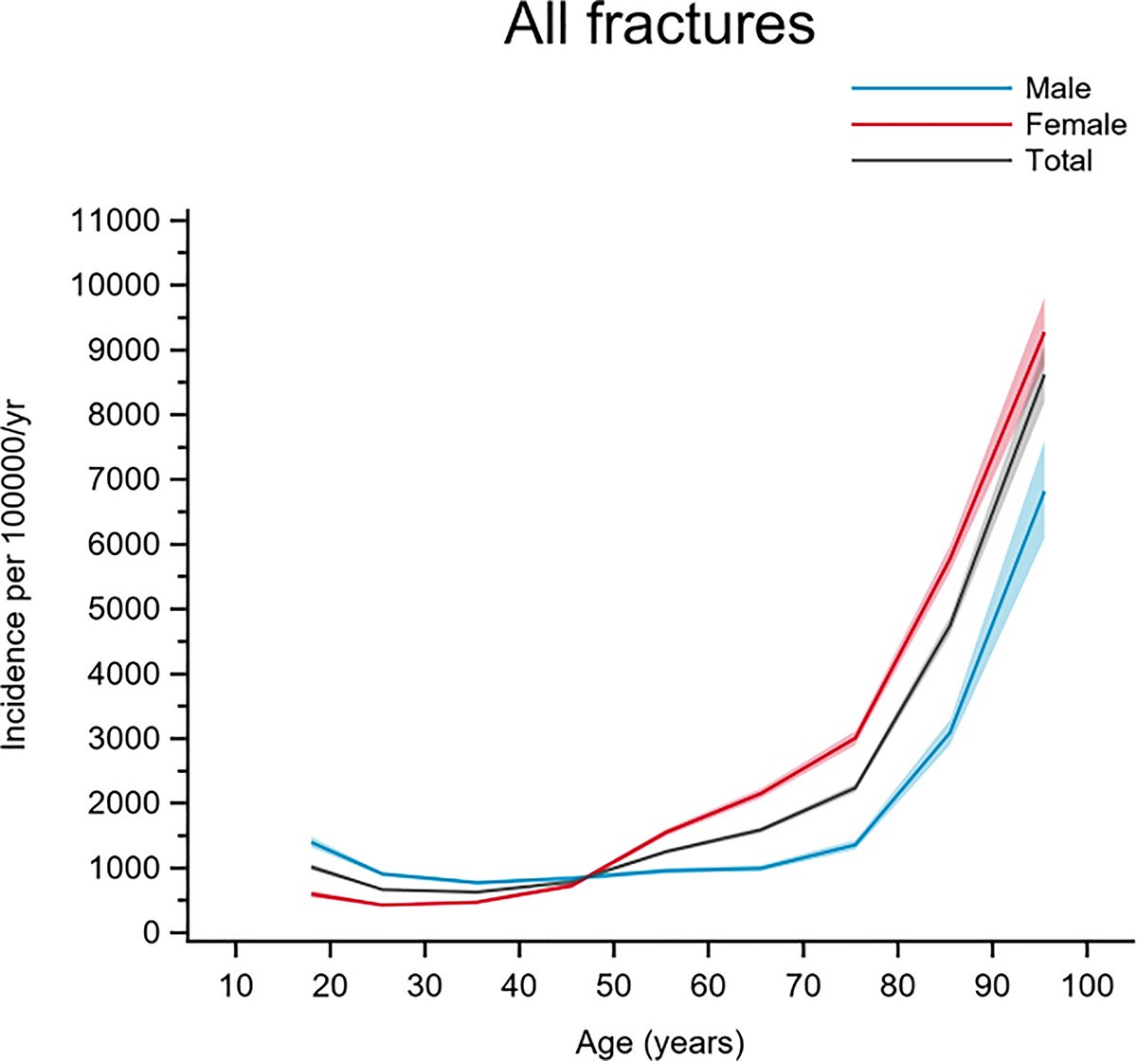
Age- and gender-specific incidence with 95% confidence interval of all fractures registered in the SFR over 4 years, 2015‒2018. Black line represents the total population; red line represents females and blue line represents males.

The age- and gender-specific incidence for suffering any type of fracture is shown in Fig 1. The overall fracture incidence was higher for men than for women until the age of 48 years, after which women had a higher fracture incidence.

The mean ages and the proportion of the fractures in individuals above 50, 65 and 75 years of age respectively for all different fracture locations for all registered patients, and for women and men separately, are given in Tables 3, 4 and 5.

**Table 3:** Numbers of all fractures, arranged in order of decreasing mean age and with proportion of each of the fracture types in patients over 50, 65, and 75 years of age.

Fracture	n	Mean age (years)	> 50 years (%)	> 65 years (%)	> 75 years (%)
Proximal femur	3,993	81.1	97.8	92.3	77.2
Pelvis	716	73.2	85.8	72.6	62.7
Femoral diaphysis	211	70.4	82.0	69.2	57.8
Distal femur	204	70.0	83.3	68.6	52.0
Acetabulum	164	68.3	80.5	63.4	47.0
Proximal humerus	2,237	66.6	84.8	59.5	34.4
Distal humerus	249	63.5	75.9	55.4	37.8
Spine	670	63.0	74.2	53.0	38.8
Humeral diaphysis	323	60.4	73.1	49.2	30.7
Patella	308	60.3	70.8	50.0	30.2
Distal radius	4,445	59.8	74.5	44.2	24.0
Scapula	304	55.2	62.5	32.2	16.1
Proximal tibia	545	55.0	62.0	32.8	19.6
Ankle	2,799	52.8	58.5	30.5	14.2
Proximal forearm	1,413	49.0	50.7	21.9	10.0
Clavicle	991	48.6	47.7	23.5	13.0
Distal tibia	217	48.2	44.7	23.5	10.1
Tibia diaphysis	291	48.0	47.4	23.0	11.7
Calcaneus	252	46.7	42.5	19.4	6.7
Metatarsal	1,339	46.4	44.8	18.4	8.5
Forearm	196	46.2	41.8	25.0	14.8
Finger phalanx	1,549	45.8	40.5	19.9	8.4
Toe phalanx	804	44.8	39.8	13.2	4.2
Carpus	606	42.7	35.8	15.7	6.9
Midfoot	283	42.1	36.0	13.1	4.2
Metacarpal	1,964	40.1	31.2	15.9	8.0
Talus	96	37.4	20.8	10.4	3.1

n, number of fractures for each fracture type; %, percentage of all fractures.

**Table 4:** Numbers of all fractures occurring in men, arranged in order of decreasing mean age, and with proportion of each of the fracture types in patients over 50, 65, and 75 years of age.

Fracture	n	Mean age (years)	> 50 years (%)	> 65 years (%)	> 75 years (%)
Proximal femur	1,350	77.9	95.8	87.1	67.9
Acetabulum	110	64.2	74.5	55.5	37.3
Pelvis	180	63.3	72.2	53.3	40.0
Proximal humerus	649	59.0	66.7	42.7	23.6
Spine	355	57.8	65.4	41.7	27.6
Distal humerus	85	56.5	65.9	43.5	27.1
Distal femur	53	55.2	66.0	45.3	18.9
Patella	131	53.0	55.0	36.6	19.1
Femoral diaphysis	57	52.6	54.4	36.8	24.6
Humeral diaphysis	147	52.1	57.8	35.4	16.3
Scapula	191	50.0	49.2	21.5	12.6
Distal radius	1,186	48.6	50.4	24.5	12.9
Ankle	1,099	46.8	45.7	20.0	7.8
Proximal tibia	231	45.8	40.7	13.4	6.9
Calcaneus	164	43.7	35.4	14.6	3.0
Clavicle	678	43.6	37.6	14.6	6.2
Distal tibia	117	43.0	34.2	14.5	2.6
Toe phalanx	357	42.0	30.3	10.4	4.8
Proximal forearm	599	41.9	31.2	12.0	5.7
Finger phalanx	851	41.8	30.9	13.0	4.5
Tibia diaphysis	163	41.7	32.5	12.3	3.7
Metatarsal	548	39.9	29.0	9.9	3.6
Midfoot	151	39.3	28.5	8.6	2.0
Carpus	425	38.4	24.5	9.2	4.2
Forearm	108	37.5	25.0	8.3	3.7
Metacarpal	1,377	34.4	18.4	7.5	3.4
Talus	61	33.3	13.1	4.9	1.6

n, number of fractures for each fracture type; %, percentage of all fractures.

**Table 5:** Numbers of all fractures occurring in women, arranged in order of decreasing mean age, and with proportion of each of the fracture types in patients over 50, 65, and 75 years of age.

Fracture	n	Mean age (years)	> 50 years (%)	> 65 years (%)	> 75 years (%)
Proximal femur	2,643	82.8	98.9	95.0	82.0
Femoral diaphysis	154	77.0	92.2	81.2	70.1
Acetabulum	54	76.6	92.6	79.6	66.7
Pelvis	536	76.5	90.3	79.1	70.3
Distal femur	151	75.2	89.4	76.8	63.6
Proximal humerus	1,588	69.7	92.2	66.4	38.9
Spine	315	68.7	84.1	65.7	51.4
Humeral diaphysis	176	67.3	85.8	60.8	42.6
Distal humerus	164	67.1	81.1	61.6	43.3
Patella	177	65.7	82.5	59.9	38.4
Distal radius	3,259	63.9	83.2	51.4	28.0
Scapula	113	63.9	85.0	50.4	22.1
Proximal tibia	314	61.7	77.7	47.1	29.0
Clavicle	313	59.3	69.6	42.8	27.8
Forearm	88	56.9	62.5	45.5	28.4
Ankle	1,700	56.7	66.8	37.3	18.3
Tibia diaphysis	128	56.1	66.4	36.7	21.9
Proximal forearm	814	54.3	65.0	29.2	13.3
Distal tibia	100	54.2	57.0	34.0	19.0
Metacarpal	587	53.5	61.3	35.8	18.9
Carpus	181	53.1	62.4	30.9	13.3
Calcaneus	88	52.4	55.7	28.4	13.6
Metatarsal	791	51.0	55.8	24.4	11.9
Finger phalanx	698	50.7	52.3	28.4	13.2
Toe phalanx	447	47.0	47.4	15.4	3.8
Midfoot	132	45.3	44.7	18.2	6.8
Talus	35	44.5	34.3	20.0	5.7

n, number of fractures for each fracture type; %, percentage of all fractures.

The five most common fracture locations showed large differences regarding mean age (81.1 years for proximal femur fractures, 66.61 years for proximal humerus fractures, 59.8 years for distal radius fractures, 52.8 years for ankle fractures, and 40.1 years for metacarpal fractures).

The mean age for fractures traditionally considered to be fragility fractures was above 63 years (both genders combined). Proximal femur fracture was the most closely associated with high age; 97.8% of individuals sustaining this fracture were 50 years of age or more and 77.2% were 75 years of age or more (Table 3).

Three of the four most common fracture locations seen in women over 50 years of age are traditionally considered to be fragility fractures (distal radius, proximal femur, and proximal humerus fractures). Other common fractures in women in this age group (> 50 years of age) were femoral diaphysis (with 92.2% occurring in the > 50-year age group), acetabulum (92.2%), pelvis (90.3%), distal femur (89.4%), and spine (84.1%). More than 50% of all these fractures occurred in individuals over 75 years of age. For proximal tibia fractures, often considered to belong to the group of fragility fractures, the relation to high age was somewhat weaker with a high proportion, 77.7%, occurring in the > 50-year age group but only 29% in the > 75-year age group. Similar proportions were seen for distal radius fractures, with 83.2% occurring in the > 50-year age group but only 28.0% in the > 75-year age group (Table 5).

The age distributions for the different fractures in men and women are given in Tables 4 and 5. Overall, the mean age for the typical fragility fractures was higher in women than in men, and a larger proportion of these fractures also occurred in women. The proportions of fractures occurring at different ages showed similar frequencies in women over 50 years of age and in men over 65 years of age, suggesting a shift in age of about 15 years between sexes for fragility fractures.

### Grouping of fractures based on the graphical patterns for age and gender incidence

**Fig 2:**
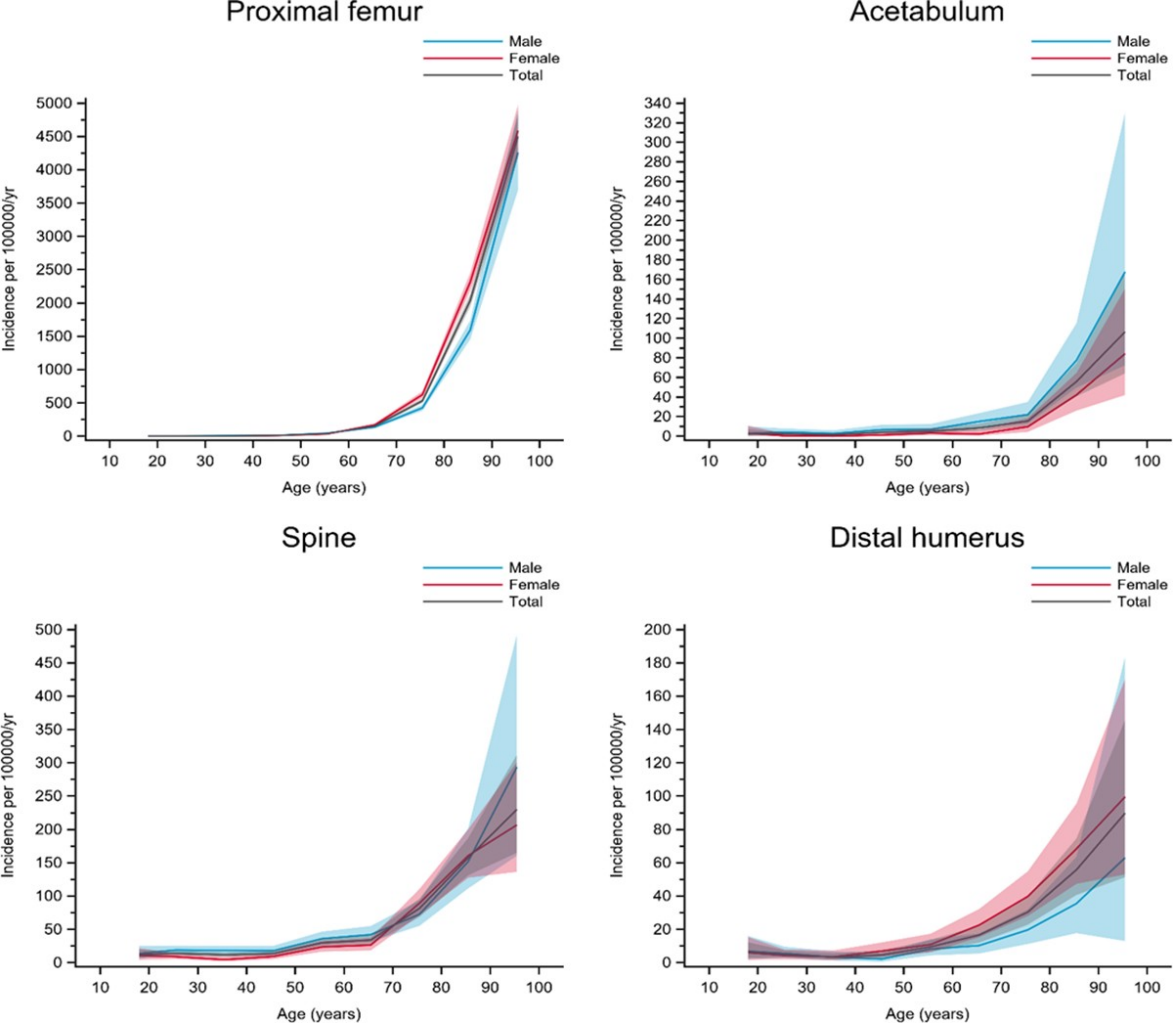
Fractures in group A. Age- and gender-specific incidence with 95% confidence interval. Black line represents the total population; red line represents females and blue line represents males.

Seven groups were created, based on the graphical patterns for age- and gender-specific incidence. The fracture incidence for the genders are displayed separately as well as the overall incidence in the graphs (Fig 2A–2G). Since the incidence of the different fracture locations varied substantially, different scales for the different curves had to be used on the y-axis.

Group A included fractures with increasing incidence with higher age, a steep increase in incidence at around 60‒70 years, and a similar pattern for both men and women. Proximal femur, acetabulum, spine, and distal humerus fractures belonged to this group (Fig 2).

**Fig 3:**
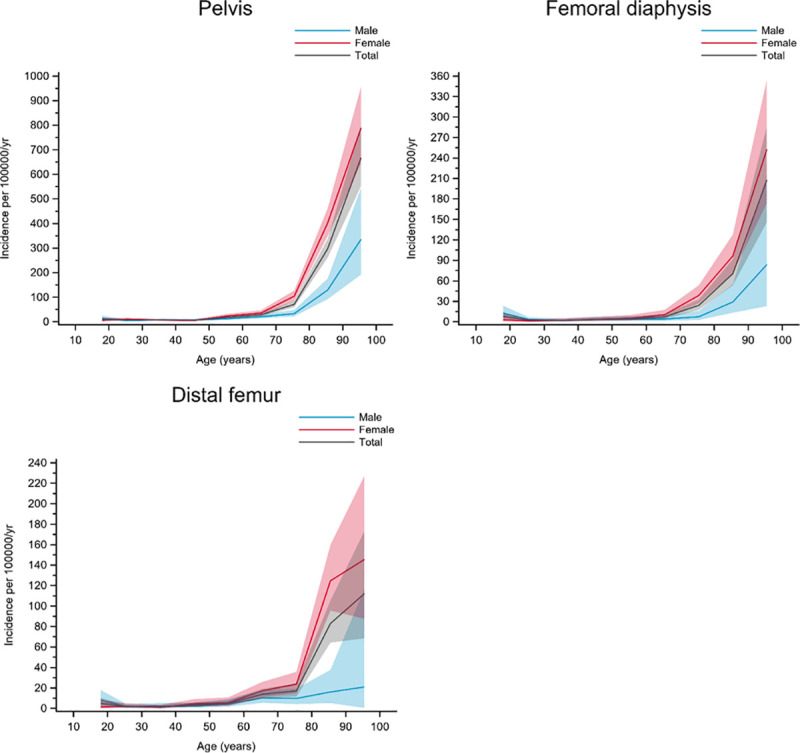
Fractures in group B. Age- and gender-specific incidence with 95% confidence interval. Black line represents the total population; red line represents females and blue line represents males.

Group B included fractures with clearly increasing incidence with age, starting at around 60‒70 years of age where women accounted for most of the increase whereas the men showed less of an increase in incidence. Pelvis, femoral diaphysis, and distal femur fractures belonged to this group (Fig 3)

**Fig 4:**
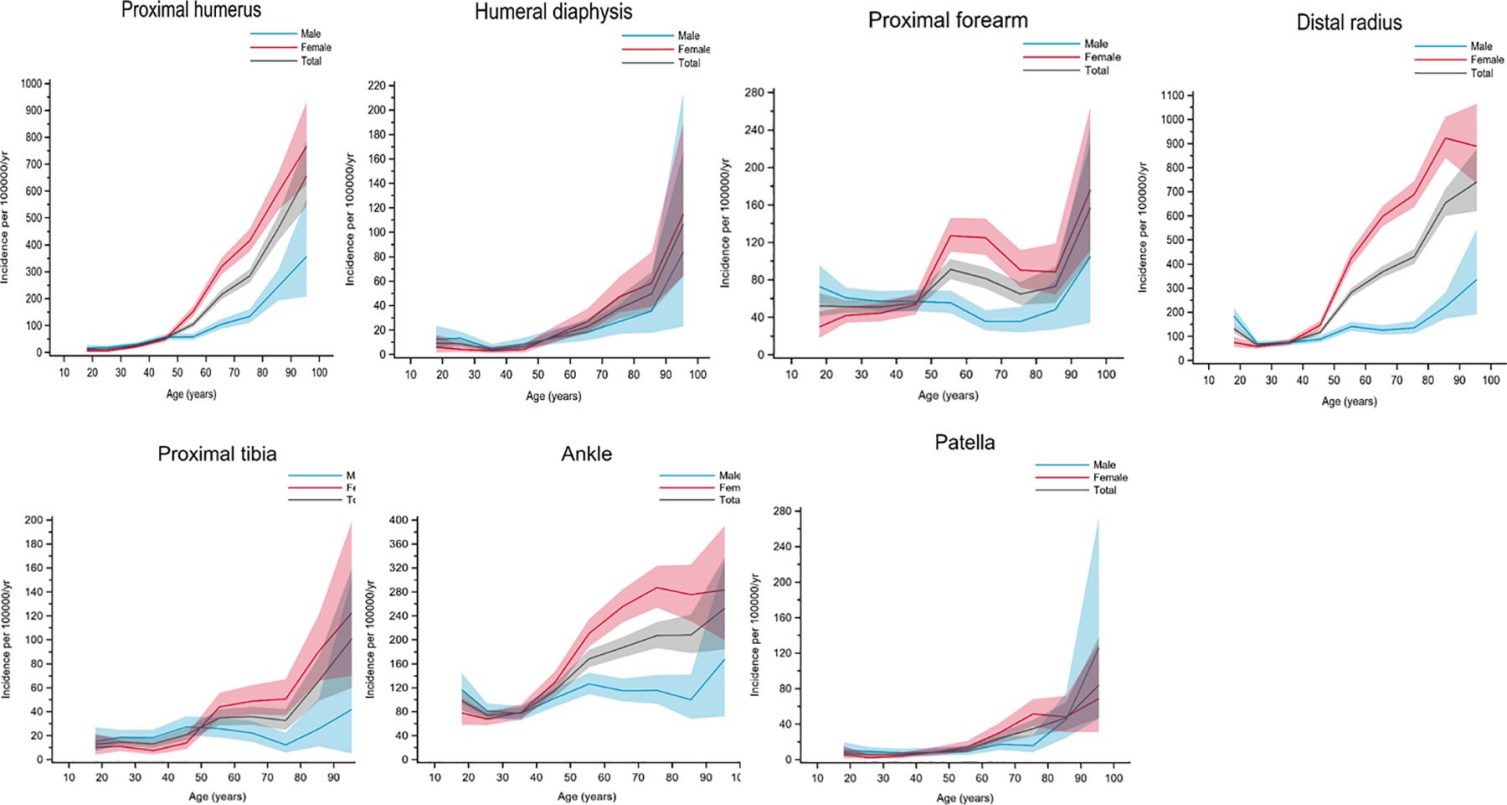
Fractures in group C. Age- and gender-specific incidence with 95% confidence interval. Black line represents the total population; red line represents females and blue line represents males.

Group C included fractures with increasing incidence with age, starting at around 40‒50 years of age. Women mostly accounted for the increase in incidence in this group. Proximal humerus, humeral diaphysis, proximal forearm, distal radius, proximal tibia, ankle, and patella fractures belonged to this group (Fig 4).

**Fig 5:**
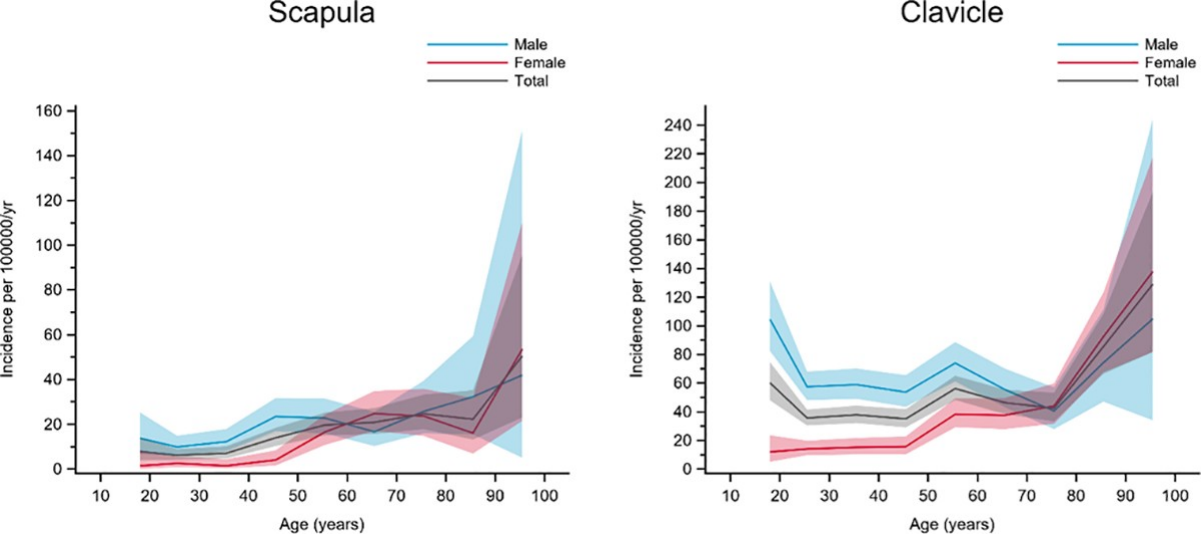
Fractures in group D. Age- and gender-specific incidence with 95% confidence interval. Black line represents the total population; red line represents females and blue line represents males.

Group D included fractures with a higher incidence in men of most ages; a higher or similar incidence in women was only seen in the very elderly. Scapula and clavicle fractures belonged to this group (Fig 5).

**Fig 6:**
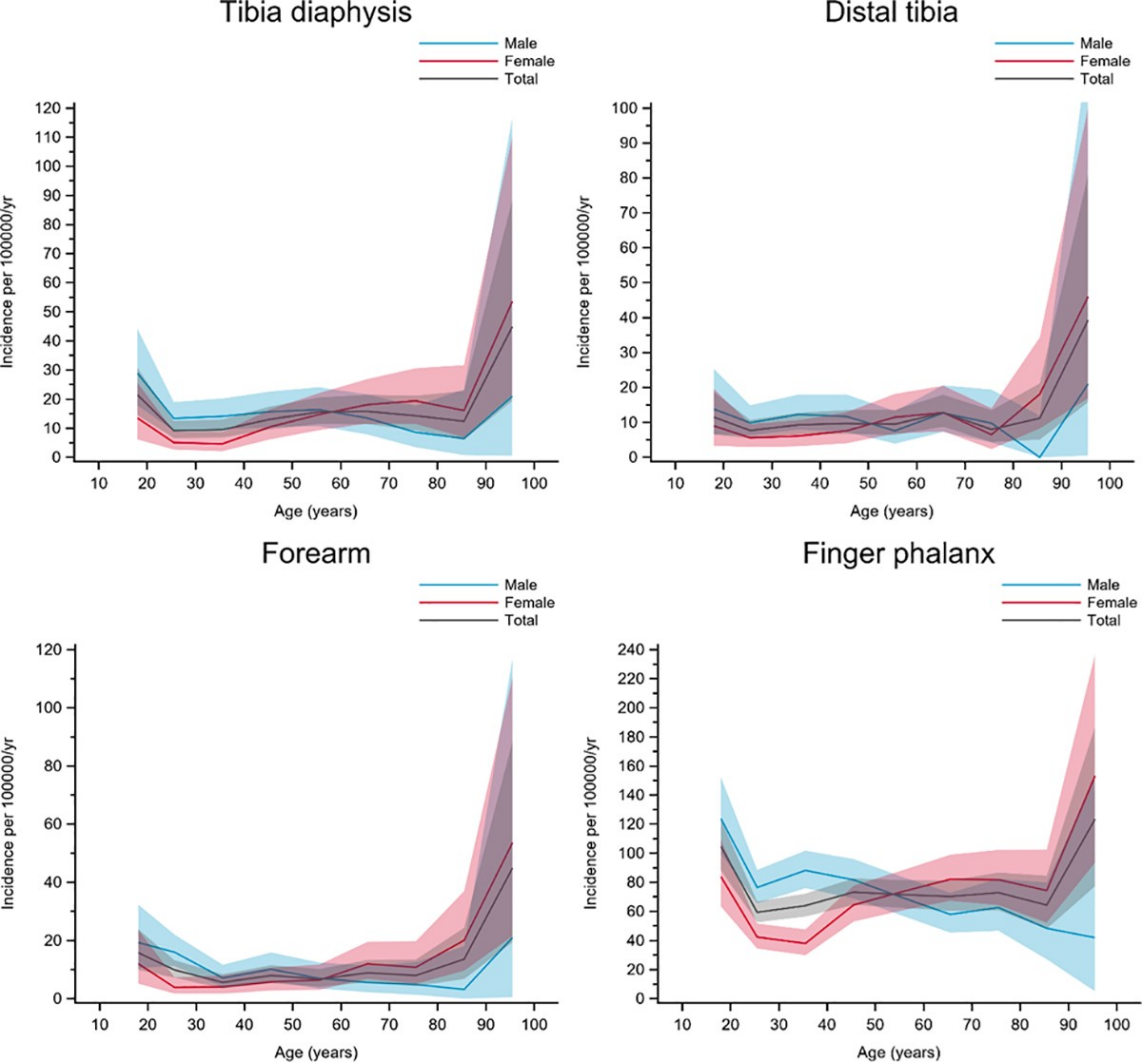
Fractures in group E. Age- and gender-specific incidence with 95% confidence interval. Black line represents the total population; red line represents females and blue line represents males.

Group E included fracture types with a higher incidence in men than in women until the age of approximately 50 years, after which there was a higher incidence in women. Except for the highest age groups (> 75 years), the incidence in men and women combined was fairly consistent at all ages―giving relatively flat overall incidence curves. Tibia diaphysis, distal tibia, forearm, and finger phalanx fractures belonged to this group (Fig 6).

**Fig 7:**
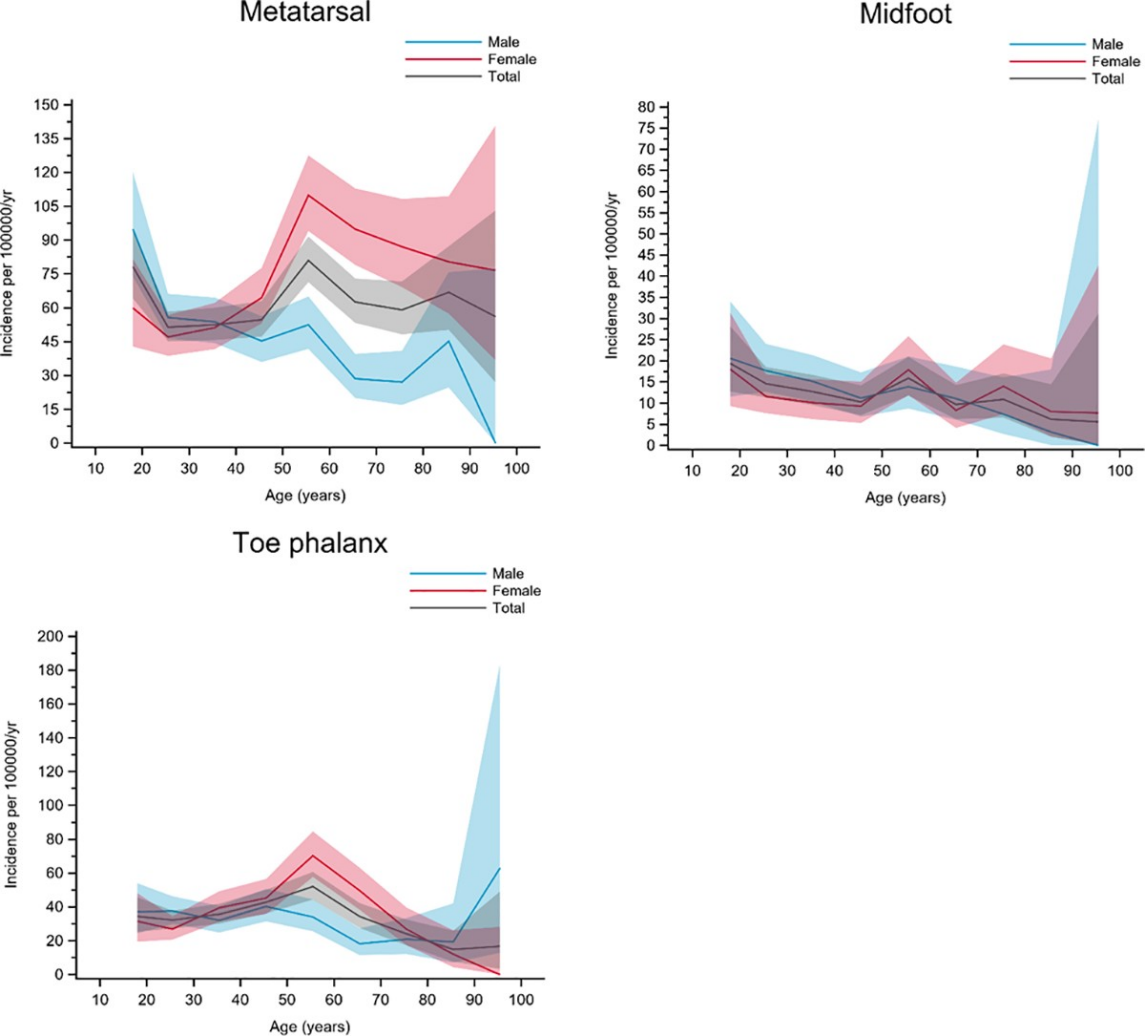
Fractures in group F. Age- and gender-specific incidence with 95% confidence interval. Black line represents the total population; red line represents females and blue line represents males.

Group F included fractures with a peak in incidence around 50 years of age. For two of the fracture types, a bimodal appearance with a smaller peak in the youngest individuals (around 20 years of age) was seen. Metatarsal, midfoot, and toe phalanx fractures belonged to this group (Fig 7).

**Fig 8:**
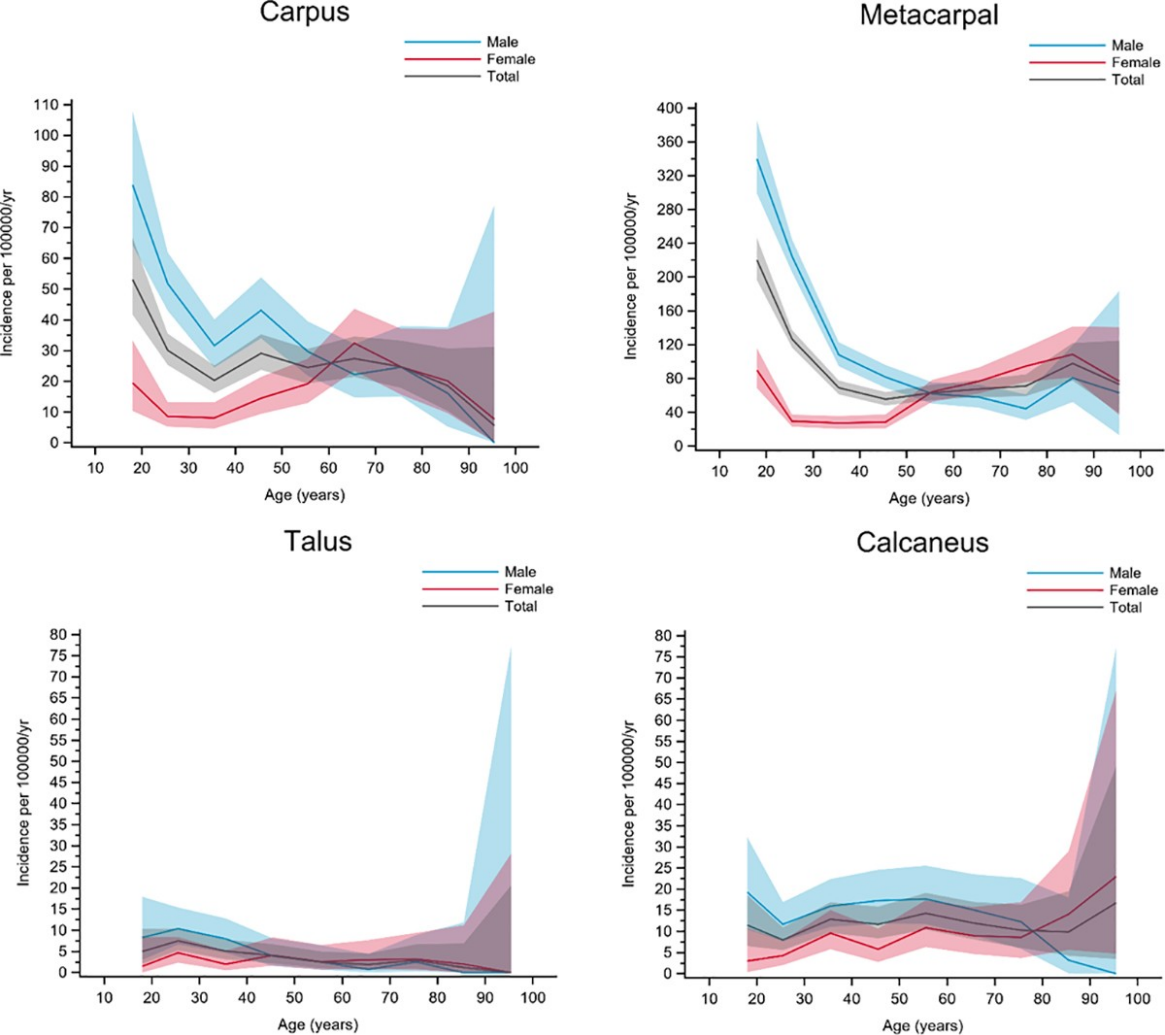
Fractures in group G. Age- and gender-specific incidence with 95% confidence interval. Black line represents the total population; red line represents females and blue line represents males.

Group G included fractures with the highest incidence observed in young men. In women, the curves were flat or gradually increased with age up to about 70 years. The carpal, metacarpal, talar, and calcaneal fractures belonged to this group, but calcaneal fractures showed a slightly different pattern (Fig 8).

## Discussion

This paper reports on incidence of all fracture locations based on prospectively collected data in a quality register. The overall fracture incidence seen in the present study was 1,229 per 100,000 individuals per year and the most common fractures were located in the distal radius, proximal femur, ankle, proximal humerus, and metacarpal bones. Fractures in these locations constituted more than 50% of the total number. The grouping of fractures based on incidence patterns, regarding age and gender, was used to present at which locations fragility fractures mainly occurs. The incidence curves clearly showed that for many more fractures, than traditionally considered as fragility fractures, elderly individuals accounted for the majority of the fractures. This fits well with that most of the fractures were labelled as caused by low-energy trauma.

Distal radius fractures was, as described in previous studies, found to be the most common fracture [5, 19, 23, 37]. Interestingly the incidences for distal radius fractures in women were 29.2 per 100,000 individuals per year in the current study and 30.2 per 100,000 individuals per year in the study by van Staa et al. [38]. These cohorts were collected 20 years apart, however, and the similarity in incidence suggests a decline in radius fracture incidence in the older population, since there has been a known increase in the proportion of elderly in the western world during this time. A true decline in radial fracture incidence for older individuals are however speculative based on the data from the present study. In a study by Dimai et al. on the population in Austria a decline of radial fracture incidence for women over 50 years of age between the years 1999–2010 has been described possibly supporting this [39]. However, differences in the data collection between the studies suggest that comparisons need to be interpreted with caution.

The frequencies of femur fractures in the present study differ substantially from those in previous studies, probably because of differences in how the fractures were classified in the different studies. For example, Driessen et al. combined all femur fractures into one group [19]―in contrast to the present study, where the femur fractures were divided into proximal, diaphysis, and distal femur fracture. The varied classification/grouping of fractures limits the possibility of comparing incidence rates between studies. Furthermore factors such as how the data on reported fractures were obtained (collected in a register or collected from hospital databases), the uncertainty about completeness in different studies, and also the fact that many studies have used relative and not absolute incidence numbers adds to the difficulty in making comparisons between studies. However, the distributions of fracture incidence in relation to age in the present study are in overall agreement with other studies [19, 23, 38].

Clear differences in incidence were observed between men and women for almost all fracture locations in the present study. One of the most obvious differences was that for all osteoporosis/osteopenia-related fractures, the time at which the incidences rapidly increased occurred at older ages in men than in women. Another clear pattern was that higher incidences regarding most fractures were observed at younger ages for men than for women. This probably reflects the fact that young men take higher risks and more often suffer high-energy trauma than young women. Overall, considering all ages and all fractures, the fracture incidence in women was higher than in men in the present study, driven by the higher incidence of osteoporotic fractures in women. An overall higher incidence in women was reported by Court-Brown et al. [37] and by Driessen et al. [19], but not in the paper by Court-Brown et al. [23].

The overall fracture incidence of 1,229 per 100,000 individuals per year in the present study is close to the incidence reported by Court-Brown et al. for the year 2000 (1,113 per 100,000 individuals per year) [23], by the same authors for 2010‒2011 (1,441 per 100,000 individuals per year) [37], and by Beerkamp et al. for 2012 (1,291 per 100,000 individuals per year) [5]. In the study by Driessen et al. from 2011, a total incidence of 1,910 per 100,000 individuals per year was found [19]. This is markedly higher than in the present study. The variation in fracture incidence between studies may depend on many factors―such as study design, source, societal differences, and age distribution―used for the data collection. The inclusion age for the different studies above varied from over 12 years of age [23], to what might be considered a skeletally mature population, above the age of 16 years [5], to over 20 years [19, 38]. Some authors have concentrated on the incidence for specific age groups; for example, in the study by Court-Brown from 2014 only patients over 35 years of age were included [37]. In the study by Beerkamp et al. with the same inclusion age (> 16 years) as in the present study and performed in a similar community, an almost identical overall fracture incidence was seen, 1,291 per 100,000 individuals per year as opposed to our 1,229 per 100,000 individuals per year, indicating that such factors play a role regarding fracture incidence [5].

The present study was inspired by previous work by Court-Brown and co-workers, who have published a number of studies on fracture incidence in the Edinburgh area [11, 23, 37, 40]. In one of their papers, Court-Brown et al. suggested a division of the different fracture types into eight groups based on the pattern of age- and gender-specific incidence [23]. In the present study, the catchment area was similar in size and in a country with similarities in socioeconomic, demographic, and climate characteristics, but the data were collected in a somewhat different way. In the study from Court-Brown et al., no incidence figures were included, only graphical illustration of the patterns. In the present study, seven groups instead of eight were constructed based on the age- and gender-specific incidence graphs. The groups in the present study did not completely match the groups constructed by Court-Brown et al. but had many similarities.

There are a number of other factors of interest when studying and describing different fractures, such as cause of injury, and dividing the trauma mechanism into high and low energy trauma. Further, whether the fractures are open or closed injuries may be of interest. However, for the fracture locations with the highest incidence and most of the fractures traditionally considered as fragility fractures the numbers of high-energy fractures as well as open fractures are low. Therefore, incidence figures on these fracture locations are probably less affected by differences in the proportion of high/low energy trauma than by gender and age. The value of classifying trauma causing a fracture into high or low energy can be debated [41]. Fractures for all patients above 50 years of age, regardless of trauma type, should be evaluated carefully and a possible underlying cause of osteoporosis/osteopenia considered.

One of the fracture types where we expect not to catch all fractures within the register are the vertebral fractures. It is well known that elderly patients do not always seek medical care and/or are referred to a radiological examination after a minor trauma. For vertebral fractures 22% were classified as related to high-energy trauma, which is probably explained by underdiagnosing osteoporotic vertebral fractures.

For some selected fracture types detailed data on cause of injury, proportions of closed/open fractures and treatments based on the SFR have been published [20, 21], however there is more to explore.

The major strength of the present study was the structured prospective data collection by a national quality register. Furthermore, the fracture classification in the SFR has been evaluated and showed adequate validity [32–35]. One limitation is that the SFR does not include fractures sustained abroad or in patients who do not have a Swedish personal identity number (although living within the catchment area). Moreover, individuals within the catchment area who sustain minor injuries that are not seen at the Accident and Emergency Department of the hospital are not registered in the SFR. This means that the true incidence might be somewhat higher than reported, especially for less significant fractures such as toe fractures and distal finger fractures. However, the vast majority of fractures that occur in the Gothenburg area are treated at Sahlgrenska University Hospital, which is the only provider of emergency care, involving fracture treatments for all fractures, in the catchment area. Further, no data on comorbidities and other factors of interest for fracture occurrence e.g. body mass index, investigations on bone quality are available in the SFR, and such data is not presently easily accessible, especially for out-patients, but may be explored by combining different registries and data bases in the future.

The age- and gender-specific fracture incidence rates for adults reported here using a defined catchment area can be expected to reasonably well reflect the incidence for the full nation of the SFR register and be relatively similar in countries with a comparable socioeconomic structure. However, this of course remains to be investigated when completeness in the SFR for the full nation is as good as for the investigated region. Further, comparison to other nations remains to be performed when similar registers are set-up.

The SFR register is today used to identify patients with probable fragility fractures (hip, proximal humerus, spine, pelvic and distal radius over the age of 50 years) for osteoporosis investigation and subsequent treatments. In a recent study on fracture liaison service programs, including data from the SFR, a reduced rate of recurrent fractures during a follow-up period of 0–6 years was demonstrated [42]. Data from the present study shows an increasing incidence with age also in some fractures traditionally not considered as fragility fractures e.g. proximal tibia and ankle (women) why these also should be considered for bone quality investigations and secondary prevention treatments.

A limitation of the present study is that only five fracture locations (some of the most common locations) are validated regarded fracture classification accuracy.

With an ageing population, certain fracture types will become more common. In young individuals, the gender difference is obvious―with men suffering fractures more often than women in the same age group. However, at older ages the fracture incidence pattern is similar for men and women, but the age at which the slope of the curve increases occurs earlier in women. This also means that for almost all fracture types, women have a higher incidence than men around 60‒70 years of age. Overall, data collected and classified prospectively by orthopaedic surgeons in a national quality register can help to increase our knowledge on fracture incidence and such data could further be used for longitudinal analyses, comparisons between different geographical areas and socioeconomic conditions.

## Conclusion

In conclusion, the incidence numbers vary substantially according to age and gender for different locations and presumably there are more fractures, than traditionally considered, that may be labeled as fragility fractures. The description of incidence curves for different fracture locations, supported by grouping of the fractures based on age and gender, can be used to provide easily understandable information on what fractures to be expected in a particular population. This may be of assistance, in healthcare planning and community-based preventive measures, especially in countries with similar socioeconomic structure and fracture burden.
